# Knowledge, enablers, and barriers to TB preventive treatment among health care workers

**DOI:** 10.5588/pha.24.0044

**Published:** 2025-09-03

**Authors:** O. Chukwuogo, O. Daniel, A. Ihesie, R. Eneogu, B. Odume, A. Agbaje, D. Nongo, J. Kuye, O. Oyelaran, W. Van Gemert, L. Mupfumi, E. Akpanowo, S. Asuke, C. D’auvergne, O. Chijioke-Akaniro, C. Anyaike, S. Olarewaju

**Affiliations:** ^1^ KNCV, Abuja, Nigeria;; ^2^ IHVN, Abuja, Nigeria;; ^3^ USAID, Abuja, Nigeria;; ^4^ John Snow, Inc., Abuja, Nigeria;; ^5^ Stop TB Partnership, Geneva, Switzerland;; ^6^ Bingham University Teaching Hospital, Jos, Nigeria;; ^7^ USAID Global Health Bureau, Washington, DC, USA;; ^8^ Federal Ministry of Health, Abuja, Nigeria;; ^9^ Department of Community Medicine, Osun State University, Osogbo, Nigeria.

**Keywords:** tuberculosis, TPT, knowledge, TBI, Nigeria, competency building

## Abstract

**BACKGROUND:**

As part of its TB control efforts, the Nigeria National TB Program has prioritised implementation of TB preventive treatment (TPT) especially among all contacts of TB patients. This study aims to assess knowledge, perceived enablers, and barriers to TPT among health care workers (HCWs) in Nigeria.

**METHODS:**

This was a cross-sectional descriptive study using mixed methods. Quantitative data were collected from 434 HCWs and analysed using SPSS version 25, and in-depth interviews were conducted on 36 purposely selected HCWs with thematic analysis.

**RESULT:**

More than half of the respondents (55.7%) had good knowledge of TPT. Nurses, doctors, and other HCWs working in public tertiary institutions had better knowledge compared with other cadres. Adequate knowledge of types of TPT regimens and belief in their effectiveness were elicited as enablers, whereas barriers included suboptimal contact tracing system, TPT stock-outs, long duration of TPT, unavailability of TB infection testing before TPT, absence of transport logistics support for patients to receive TPT, and poor HCW capacity.

**CONCLUSION:**

HCWs in public tertiary settings had better knowledge of TPT. Successful scale-up of TPT services requires competency building for other cadres and interventions addressing other identifiable barriers.

TB continues to be a significant cause of morbidity and mortality globally, and one quarter of the world’s population has TB infection (TBI).^1^ According to the WHO, in 2022, 10.6 million people developed TB, while 1.3 million passed away before they could access treatment.^2^ Nigeria’s estimated burden of TB ranks sixth in the world among high-burden countries and first in Africa, with a case notification rate of 282,184 in 2022.^2^ The likelihood of TBI rises with increasing contact with TB patients. Approximately 5%–10% of infected individuals will develop TB in their lifetime, most within 5 years post-infection.^3^ However, one of the targets of sustainable development goal 3 of eliminating TB disease by 2030 requires identification of people at risk of TBI, diagnosis, and treatment with TB preventive therapy (TPT) before the disease develops. This is part of the WHO’s initiative towards reducing risk of progression of TBI to TB disease and was prioritised in recent years by the National TB Program (NTP) for beneficiaries (mainly people living with HIV as well as contacts of index TB patients). Previous studies observed inadequate knowledge, poor perception, and skills, as well as misconceptions about TPT^4^ among health care workers (HCWs) as barriers to successful TPT implementation among under-five contacts and HIV-positive individuals placed on TPT.^5–8^ The WHO introduced newer short-course TPT regimens as part of measures towards achieving one of the 2018 UN High-Level Meeting targets on TB, protecting 30 million individuals from having TB disease by providing them with TPT by 2022.^9^ The introduction of such a new approach to improve TPT implementation requires collective efforts and investment from governments and stakeholders. This includes donors to strengthen the health system across the cascade of care for TB and enhance provider and community awareness, knowledge of importance of TPT, as well as improved access to new tools for detecting TBI and shorter TPT regimens to meet set targets.^3^ In response, the 3HR shorter regimen for TPT was introduced by the NTP of Nigeria with support from USAID under the Stop TB Partnership introducing New Tools Project for eligible adult and child contacts. This study seeks to assess HCWs’ knowledge of TPT among contacts of TB patients and the perceived enablers and barriers to TPT for contacts of TB patients in Nigeria.

## METHODS

This was a descriptive cross-sectional study conducted among consenting HCWs in public and private health facilities in selected USAID TB-LON-supported facilities across 18 states in Nigeria. A sample size of 457 was calculated for the quantitative survey based on a previous study.^10^ Inclusion criteria included HCWs who expressed willingness to participate while those not available at the time of survey were excluded. A multi-stage sampling technique was adopted to recruit 457 HCWs for the quantitative survey based on proportionate allocation, and 36 participants were purposely selected for the qualitative study across 18 implementing states.

### Data collection

A pre-tested questionnaire with 30-item questions related to knowledge and barriers to TPT adapted from a previous study was administered by trained research assistants.^11^ The independent variables included socio-demographic characteristics of HCWs, whereas the dependent variables included HCW’s knowledge of TPT. Respondents’ answers were scored with 1 for ‘correct’ and 0 for ‘incorrect’ or ‘don’t know’ responses. Total and mean scores were computed. Those who scored greater than or equal to the mean knowledge score were categorised as having ‘Good knowledge’, while those who scored less than the mean were classified as having ‘Poor knowledge’.^11,12^ Qualitative data were collected using an in-depth interview (IDI) guide from 36 HCWs on their perception of facilitators and barriers to TPT. All face-to-face interviews were conducted in English (or the local language, using a translator) and responses were hand-written and recorded for subsequent transcription by the interviewer. Quantitative data were analysed using SPSS software version 25 after cleaning through descriptive statistics (frequencies and percentages) and inferential statistics using appropriate test statistics. The significance level was set at *P* less than 0.05. Thematic analysis using a realist method was used for the qualitative data from the IDI to generate themes on facilitators and barriers to TPT. The themes were then categorised and reported using consolidated criteria for reporting qualitative research.^13^

### Ethical statement

Ethical approval for this study was obtained from the National Health Research Ethics Committee of Nigeria (NHREC). Also, the head of TB-LON facilities provided permission for the study, and each contact was asked to consent by signing the consent form to participate in the study. The consent form included an explanation of the study, its objectives, potential benefits and risks, and contact information for the study’s Principal Investigator.

## RESULTS

The majority of the HCWs interviewed (33.8%) were employed in public primary health centres (PHCs), and community health workers constituted 30.8% of the participants. Most HCWs (81.2%) had been trained on TB, with 61.2% reporting being trained on patient counselling, patient care/management, and active case finding, while 41.4% of the respondents had been trained on TPT. In terms of activities involved in TB programmes, two thirds (i.e., 63.3%) are directly involved in the provision of TPT, whereas 5.5% were adherence counsellors and referral officers (https://doi.org/10.6084/m9.figshare.29371442). The assessment of knowledge of TPT including knowledge of TBI, clinical and diagnostic evaluation methods, eligibility criteria for TPT, and TPT regimens showed that more than half of the respondents (255 [55.7%]) had good knowledge, whereas 44.3% had poor knowledge based on the mean score (https://doi.org/10.6084/m9.figshare.29371442).

Perceived barriers reported by HCWs included a lack of understanding of TB contact screening by caregivers (80.3%), refusal to take prescribed TPT (70.1%), reported transport difficulties (52.8%), preference for pharmacy or chemist shops as screening points (41.7%), stock-out of TPT drugs (22.1%), feeling of extra workload for HCWs (15.5%), stigma (10.5%), and fear of contact developing drug-resistant TB (9.2%). Table 1 shows that type of facility, employment status, years working in the TB programme, and previous training in the TB programme were associated with TPT knowledge. Among these associations, the type of facility (*P* = 0.003) and employment status (*P* = 0.034) were statistically significant. Table 2 shows that respondents who worked in public tertiary institutions were seven times more likely to have good knowledge of TPT than those who worked in the community settings (adjusted odds ratio [AOR] = 7.376, *P* = 0.020, confidence interval [CI] = 1.373–39.627). Also, respondents who were nurses were two times as likely to have good knowledge of TPT compared with respondents who had other professions in the facility (AOR = 1.870, *P* = 0.003, CI = 1.051–3.328).

**TABLE 1.** Association between background information and knowledge on TPT.

Variable	Knowledge		Significance
	Good knowledge, n (%)	Poor knowledge, n (%)	
Type of facility			
Public – tertiary health facility	58 (70.7)	24 (29.3)	X^2^ = 16.247
Public – secondary health facility	72 (52.2)	66 (47.8)	*P* = 0.003
Public – primary health facility	77 (49.7)	78 (50.3)	
Private hospital	46 (62.2)	28 (37.8)	
Community	2 (22.2)	7 (77.8)	
Employment status			
Doctor	13 (56.5)	10 (43.5)	X^2^ = 12.024
Nurse	78 (69)	35 (31)	*P* = 0.034
Health educator/counsellor	12 (44.4)	15 (55.6)	
Community health worker	72 (51.1)	69 (48.9)	
Community volunteer	28 (50)	28 (50)	
Others (contact tracing officers, health care assistants)	52 (53.1)	46 (46.9)	
Years working in TB programme			
Less than 5 years	127 (54)	108 (46)	X^2^ = 0.522
Above 5 years	128 (57.4)	95 (42.6)	*P* = 0.470
Previous TB training			
Yes	211 (56.7)	161 (43.3)	X^2^ = 0.874
No	44 (51.2)	42 (48.8)	*P* = 0.350

**TABLE 2.** Predictors of good knowledge of HCWs on TPT.

Variable	*P* value	AOR	AOR 95% CI
Type of facility			
Tertiary health facility	0.020	7.376	1.373–39.627
Secondary health facility	0.164	3.234	0.620–16.871
Primary health facility	0.144	3.378	0.661–17.275
Private hospital	0.071	4.783	0.873–26.224
Community (Ref)			
Employment status			
Doctor	0.823	0.898	0.349–2.311
Nurse	0.033	1.870	1.051–3.326
Health educator/counsellor	0.555	0.770	0.324–1.833
Community health worker	0.890	0.963	0.565–1.642
Community volunteer	0.939	1.028	0.511–2.067
Others (Ref)			

HCWs = health care workers; TPT = TB preventive treatment; AOR = adjusted odds ratio; CI = confidence interval.

### Thematic analysis of qualitative data

Of the 36 HCWs interviewed, the majority were female (52.8%), were within the age group of 36–46 years (47.2%), worked in secondary health facilities (63.9%), were directly observed treatment (DOT) providers (44.4%), and had more than 5 years of working experience (61.1%). The reported enablers and barriers of TPT implementation included themes centred around factors related to HCWs, clients, service delivery, and TPT drugs, as described in Figure.

**FIGURE.**
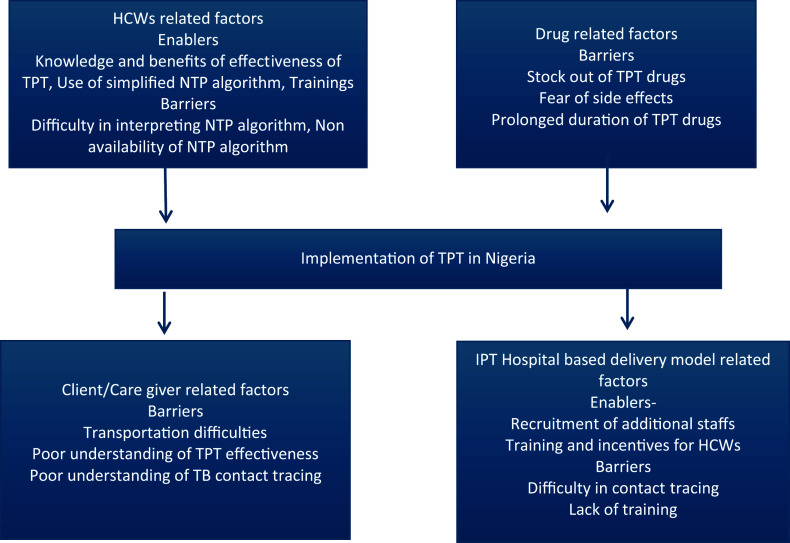
Framework on reported perceived barriers and enablers to TB preventive treatment among contacts of TB patients and health care workers’ opinion in Nigeria.

### Perceived enablers of TPT implementation

#### HCW-related factors

The majority of HCWs interviewed reported adequate knowledge of different types of TPT regimens and belief in their effectiveness, safety, and efficacy in preventing active TB, as an enabling factor towards TPT implementation:

I know of rifampicin and isoniazid. We use the one (isoniazid) that is given for six months.

(Delta HCW)

This adequate knowledge according to HCWs is linked to usage of educational tools such as the NTP TPT algorithm provided as well as training received.

It is nice, educative and it fastens active case searches in the community. However, new workers need to be trained and the old workers need to be retrained because TB is something that needs everyday training.

(Anambra HCW)

### Perceived barriers to TPT implementation

#### HCW-related factors

Some HCWs expressed difficulty in the usage and interpretation of the NTP algorithm, most especially junior staff cadres, and its non-availability as a barrier to implementation of TPT in some health centres:

It (NTP algorithm) is not easy to understand and use. It needs more explanations.

(Plateau HCW)

#### Client/caregiver-related factors

Lack of transportation for those who have to go to health care facilities for TPT drug collection, lack of understanding of TB contact screening, and ignorance of the index TB cases on the effectiveness of TPT for both children and adult contacts were reported as barriers to implementing TPT by some HCWs:

There should be provision of transport fare for the contacts, awareness of TPT, and health talk about TPT to the contacts.

(Ogun HCWs)

You don’t have TB, so why should you use drugs? I have TB, which is why I am using the drug.

(Anambra HCW)

#### Drug-related factors

Some HCWs reported challenges with stock-out of TPT, fear of side effects, and prolonged duration of TPT as part of drug-related factors hindering effective provision of TPT services.

Failure to prescribe TPT can only happen when the drug is out of stock in the facility.

(Nasarawa HCW)

It will be good if the duration of the drug regimen can be reduced.

(Oyo HCW)

The duration of the drug regimen is too long; it should be reduced, and the drug dosage too is much.

(Rivers HCW)

I think getting a drug that can last for a shorter period will make it perfect.

(Anambra HCW)

#### Hospital service delivery of TPT

Some HCWs also expressed their concern with the difficulty experienced with contact tracing, thereby requesting for recruitment of additional HCWs, training, and increase in their wages for better efficiency and delivery of TPT to TB contacts.

Those in charge should try and increase the stipends to encourage active TB contact finding.

(Nasarawa HCW)

Yes, more human resources are needed for service delivery, training and retraining of health workers on TPT.

(Kaduna HCW)

## DISCUSSION

This study assessed knowledge, enablers, and barriers to TPT among HCWs in Nigeria. TB is a disease of the poor that is common among individuals living in hard-to-reach areas where there are no specialist or tertiary institutions. We found the majority of HCWs were community health workers domiciled in PHCs. This is not surprising because Nigeria’s health policy is to establish PHCs to achieve universal access to health services, especially among the poor or marginalised population. In terms of knowledge, a little more than half (55%) of the health care providers had good knowledge of TPT. This was higher than the 43% documented in a study on knowledge of TPT done among medical doctors in South Africa in 2018 and 30.8% among general practitioners in Indonesia.^14,15^ The fact that more than three quarters of HCWs (81.3%) had been previously trained on TB, with about half specifically trained on TPT, is most likely what contributed to their good knowledge. Also, there is variation in knowledge level as workers in PHCs were less knowledgeable, similar to other findings.^16–20^ This could be due to ease of access to continuous medical education programmes provided in the tertiary institutions.

The quantitative survey revealed that a significant proportion of HCWs believed in its effectiveness and were interested in additional training. Identified programmatic barriers from the qualitative reports range from non-availability of the NTP TPT algorithm to its complexity of interpretation among junior staff. Henceforth, subsequent training on TPT should incorporate a simplified TPT as a part of educational tools to be distributed among HCWs in supported facilities. Junior cadre staff working in public and private health settings should be prioritised in subsequent training plans because those in public tertiary institutions were 86 times less likely to have good knowledge when compared with others. This finding is consistent with findings among general practitioners in Indonesia.^21^

Barriers identified were divided into four main themes, which include problems identified within the health workforce, barriers related to TPT medicine, client-related barriers, and barriers related to TPT service delivery. Regarding the health workforce challenge, respondents itemised staff shortages, inadequate knowledge of TB contact screening, and limited access to TPT policy documents. Client-related barriers included lack of access to care because of difficulties with transportation, lack of knowledge on the efficacy of TPT, and the absence of a demonstrable infection or disease to justify taking TPT, which could be related to a lack of TBI testing before TPT. Concerns about TPT medication included reports of TPT shortages and the prolonged duration of the 6-month TPT regimen. Many HCWs in this study reported the 6-month course for TPT as being too long and affecting adherence, which is similar to a qualitative study from Kenya among HIV-infected children.^22^ The newer short-course TPT regimen (1 month and 3 months) recommended by WHO for all contacts is expected to promote uptake and adherence.^23^ It is also important that service delivery models are adapted to enhance TPT uptake and adherence. The barrier presented by TPT drug stock-outs was also documented in another study in Ebonyi State, Nigeria.^10^ Other notable barriers included lack of access to care because of difficulties with transportation, inadequate training received by HCWs in initiating TPT and screening for TB, as well as the refusal of contacts to take TPT even though it is freely provided within service delivery points. These were similar to findings in other developing countries.^2,24,25^ Hence, there is a need for continuous TPT health education in the form of counselling among patients and caregivers on TPT effectiveness and efficacy.

Our study had several advantages. The mixed-method study design among a heterogamous population of HCWs across different levels of health care in 18 states provided a comprehensive perspective on knowledge, enablers, and barriers to TPT and makes the findings more generalisable. However, the cross-sectional descriptive nature limits our ability to allow for causation. Future research should assess prescription patterns, uptake, and completion of TPT.

## CONCLUSION

The HCWs surveyed demonstrated good knowledge and belief in TPT, especially among the highest cadres, such as nurses and doctors who worked in public tertiary institutions. Identified barriers included a knowledge gap among lower cadre staff that work in community settings, ignorance on the part of patients, unavailability of TBI testing before TPT, and stock-out of TPT drugs. Hence, targeted interventions should be deployed to address these barriers as TPT implementation is scaled up.
